# Gut microbiota‐stem cell niche crosstalk: A new territory for maintaining intestinal homeostasis

**DOI:** 10.1002/imt2.54

**Published:** 2022-09-27

**Authors:** Ning Ma, Xiyue Chen, Lee J. Johnston, Xi Ma

**Affiliations:** ^1^ State Key Laboratory of Animal Nutrition, College of Animal Science and Technology China Agricultural University Beijing China; ^2^ West Central Research & Outreach Center University of Minnesota Morris Minnesota USA

**Keywords:** intestinal disease, intestinal stem cell, microbiota, niche, postbiotic

## Abstract

Intestinal epithelium undergoes rapid cellular turnover, relying on the local niche, to support intestinal stem cells (ISCs) function and self‐renewal. Research into the association between ISCs and disease continues to expand at a rapid rate. However, the detailed interaction of ISCs and gut microbes remains to be elucidated. Thus, this review witnessed major advances in the crosstalk between ISCs and gut microbes, delivering key insights into (1) construction of ISC niche and molecular mechanism of how to jointly govern epithelial homeostasis and protect against intestinal diseases with the participation of Wnt, bone morphogenetic protein, and Notch; (2) differentiation fate of ISCs affect the gut microbiota. Meanwhile, the presence of intestinal microbes also regulates ISC function; (3) microbiota regulation on ISCs by Wnt and Notch signals through pattern recognition receptors; (4) how do specific microbiota‐related postbiotics influence ISCs to maintain intestinal epithelial regeneration and homeostasis that provide insights into a promising alternative therapeutic method for intestinal diseases. Considering the detailed interaction is still unclear, it is necessary to further explore the regulatory role of gut microbiota on ISCs to utilize microbes to alleviate gut disorders. Furthermore, these major advances collectively drive us ever closer to breakthroughs in regenerative medicine and cancer treatment by microbial transplantation in the clinic.

## Highlights

- Intestinal stem cells (ISCs) are protected by their niche and are regulated by Wnt, bone morphogenetic protein, and Notch.

- ISCs and their relationship with intestinal microbiota provide a feasible pathway to alleviate intestinal diseases.

- Diverse bacteria‐related postbiotics regulate ISCs and maintain their homeostasis.

## Graphical abstract

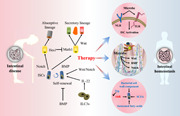

## INTRODUCTION

Intestinal stem cells (ISCs) can differentiate into intestinal epithelial cells (IECs) and enteroendocrine cells (EECs), which play a central role in governing the rapid and perpetual renewal of the mammalian intestinal epithelium [1, 2, 3]. The intestinal epithelium consists of villi and crypts. ISCs that reside at the bottom of crypts direct the consequent self‐renewal of the intestinal epithelium. The function of ISCs closely depends on the microenvironment in which they reside. The microenvironment is referred to as the ISC niche. Identification of ISC markers, isolation and in vitro culture of organoids, and use of transgenic models have contributed remarkably to our understanding of ISC self‐renewal and differentiation. Therefore, expanding our understanding of the regulatory mechanism that determine the fate of ISCs is critically important. Many immune and cell‐related diseases, such as inflammatory bowel disease (IBD) and irritable bowel syndrome (IBS), occur when being affected by a wide range of exogenous and endogenous factors [4]. Although the precise etiology of IBD and IBS remains unclear and controversial, it is clear that activated and accelerated ISC proliferation plays a central role in the recovery of intestinal integrity [5, 6]. Therefore, regulating ISC proliferation and differentiation mediated by Wnt, bone morphogenetic protein (BMP), and Notch signals is important for the repair of gut injury.

Unlike other stem cells, ISCs are continuously in contact with intestinal microbes [7]. Understanding of how Notch, Wnt, and BMP signals regulate ISCs biology is increasing but little is known about the effect of microbes on ISC function. The intestinal microbiota, together with a diverse array of metabolites, interact closely with ISCs and govern the development and turnover of stem/progenitor cells [8, 9]. However, the detailed mechanism of this interaction remains unknown.

In the complex ecosystem of the intestine, a dense and diverse community of microbiota plays a central role in several key physiological functions of the host, including, but not limited to epithelial maturation, immune system stimulation, and metabolic homeostasis. These responses are triggered by direct cell‐to‐cell interactions or by metabolites generated locally or elsewhere in the body [10]. Yet, the manner in which specific microbiota‐derived signals directly influence ISCs remains unknown. This review intends to shed light on ISCs niches and the crosstalk between ISCs and gut microbes. The major advances of ISCs and gut microbiota collectively drive us ever closer to breakthroughs for potential targets of intestinal disease intervention.

## ISC NICHE AND CELL FATE DIFFERENTIATION

### ISC niche: New insights into ISCs

ISCs are multipotent cells with a capacity for self‐maintenance, self‐renewal, and multidirectional differentiation. Many ISC populations follow a pattern of rapidly proliferative ISC divisions that are marked by leucine‐rich repeat‐containing G protein‐coupled receptor 5 (Lgr5). These cells are short‐lived and replace each other in a stochastic manner called “neutral drift” [11]. Lgr5^+^ ISCs are located in the middle of Paneth cells (PCs) at the bottom of the crypt. Wnt ligand signaling will be amplified when R‐spondin (Rspo) bind to the Lgr5 receptor [12, 13]. Wnt is necessary to maintain intestinal homeostasis. Moreover, Rspo is essential for the maintenance of ISCs and crypts [11]. In addition to crypt‐base columnar cells (marked by Lgr5), +4 cells, marked by B cell‐specific Moloney murine leukemia virus integration site 1 (Bmi1), have been described as another candidate stem cell population [14]. This slow cycling reserve crypt stem cell population has a shortage of canonical Wnt signals to modulate differentiation [15]. Both types of ISCs possess the capacity to differentiate into mature gut epithelial cells and may also be interconverted under certain conditions.

**Figure 1:**
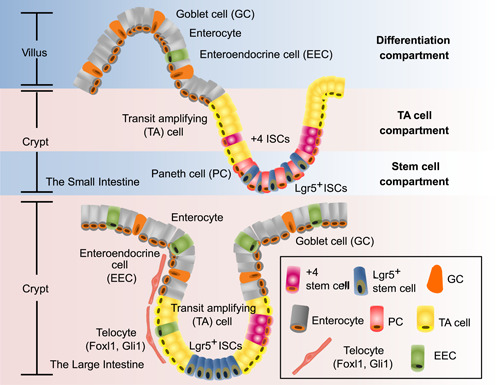
Intestinal niche: microenvironment for ISC survival. Tissue‐resident stem cells generate numerous cells to maintain and renew the small and large intestine throughout the life cycle. In the small intestine, the niche can be roughly divided into three compartments: differentiation‐, TA cell‐, and stem cell‐compartments. Lgr5^+^ ISCs are located at the bottom of the crypt with PCs, while +4 cells stay right above the PCs. In the large intestine, villi are missing and crypts located at the base of the intestinal structure become the main location of ISCs. Besides proliferating TA cells, Lgr5^+^ stem cells, and so on, there is also a specialized nonepithelial cell that induces the Wnt signaling. This kind of cell is identified as telocyte, which is characterized to generate Foxl1 and Gli1. ISCs, intestinal stem cells; Lgr5, leucine‐rich repeat‐containing G protein‐coupled receptor 5; PC, paneth cell; TA, transit amplifying.

ISCs can differentiate into transit amplifying (TA) cells for various cell lines and also proliferate and regenerate to guarantee normal epithelial turnover and tissue regeneration in the case of injury [16]. ISCs differentiate into diverse cell types of absorptive lineage or secretory lineage (PCs, goblet cells (GCs), and EEC) [17] under a complex regulatory mechanism, and have extensive contact with gut microbes, affecting microbe vitality and colonization. At the bottom of each crypt, these multiple ISCs are able to complete self‐renewal by generating early progenitor cells [18]. TA cells divide rapidly, migrate upward, and further differentiate. Enterocytes, GCs, and EECs continue to move upward toward villi and shed into the gut lumen after apoptosis, whereas PCs move downward and reside on the base [19] (Figure 1).

### Signaling pathways that regulate ISC proliferation and differentiation in the niche

**Figure 2:**
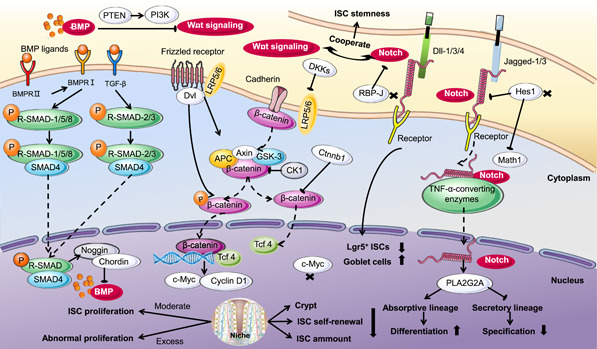
Pattern and signal transduction of ISC proliferation and differentiation. The normal operation of the ISC niche is achieved by the regulation of several signaling pathways. The Wnt, BMP, and Notch signal molecules comodulate ISC functions. Moderate activation of the Wnt signaling pathway induces ISC proliferation and self‐renewal; however, intestinal adenomas can also be caused due to abnormal proliferation resulting from excessive signaling. The BMP signaling pathway negatively regulates self‐renewal, replication, and proliferation of ISCs. Its activity is strictly controlled by cascade regulation of BMPRII, BMPRI, TGF*β*, R‐SMAD, and SMAD4. Activation of the Notch signaling pathway is decisive in promoting ISC proliferation and restricting ISC specification. This determination of ISC differentiation fate is achieved by increasing absorptive lineage differentiation while inhibiting secretory lineage specification. Receptors, membrane‐bound ligands (Jagged1,3 and Delta‐like1,3,4), and DNA binding proteins constitute the main component of the Notch. TNF‐*α*‐converting enzymes, Hes1, Math1, and RBP‐J contribute a lot to this regulation. PTEN mediates suppression of BMP to Wnt through the PI3K signaling pathway. Meanwhile, Wnt cooperating with Notch plays an important role in maintaining ISC stemness. APC, adenomatosis polyposis coli; BMP, bone morphogenetic protein; BMPR, bone morphogenetic protein receptor; c‐Myc, cellular Myc; Dkk1, Dickkopf‐related protein 1; Hes1, hairy and enhancer of split 1; ISC, intestinal stem cell; LRP6, lipoprotein receptor‐related protein 6; Math1, mouse atonal homolog 1; PI3K, phosphatidylinositol 3‐kinase; PTEN, phosphate and tension homology deleted on chromosome 10; RBP, recombination signal binding protein; R‐SMAD, receptor‐associated SMAD; SMAD4, drosophila mothers against decapentaplegic protein; TGF*β*, transforming growth factor *β*; TNF‐*α*, tumor necrosis factor alpha.

The normal function of ISCs depends on a supportive microenvironment [14] that consists of PCs, intestinal subepithelial myofibroblasts, and intestinal stromal cells. Moreover, the normal operation of this microenvironment is regulated by multiple factors, including gut microbes, enteral nutrition, the endocrine system, and several signaling pathways [20]. The Wnt, BMP, and Notch signal molecules from these cells coregulate the function of ISCs (Figure 2) [21].

#### Wnt signaling pathway induces ISC proliferation and self‐renewal

Wnt is the primary signaling pathway that drives ISC proliferation and is responsible for decreasing ISC differentiation in the niche. In this process, the activity of Wnt gradually decreases along the crypt‐villus axis and reaches a minimum at the villus, which is the exact site for differentiation [22]. This function is performed due to the continuous regulation of Wnt on the adenomatosis polyposis coli (Apc) destruction complex and *β*‐catenin. The Apc destruction complex in the cytoplasm is composed of Apc, axis inhibition 2, casein tyrosine kinase 1, and glycogen synthase kinase‐3 beta (GSK‐3*β*). The Wnt signaling pathway breaks down the Apc destruction complex by binding to frizzled target on the cell surface with a low abundance of lipoprotein receptor‐related protein (LRP) 5/6. The decomposed Apc destruction complex in the cytoplasm subsequently breaks down phosphorylated *β*‐catenin to translocate it into the nucleus where *β*‐catenin combined with T‐cell factor 4 (Tcf4) stimulates target genes that activate ISCs (Figure 2) [23].

Activation of the Wnt signaling pathway indicates its importance in regulating epithelial homeostasis. The activated Wnt signaling pathway has been identified in a majority of colorectal cancers with Apc gene mutations [24]. In rare cases of Apc‐positive colorectal cancers, mutation of *β*‐catenin, which is a pivotal upstream effector of the Wnt signaling pathway, has also been observed [25].

The Wnt signaling pathway is essential for establishing the ISC compartment in the postnatal state. Dickkopf‐related protein 1 (Dkk1) serves as an inhibitor of the Wnt signaling pathway in the intestine and its ectopic expression reduces the number of ISCs and proliferative cells in adult mice [26]. Dkk1 reduced ISC compartmentality and impaired ISC self‐renewal [27], a response similar to that observed with intestinal‐specific knockout of Ctnnb1 (encodes *β*‐catenin) [28]. Considering that Tcf4 is affected by *β*‐catenin in the Wnt signaling pathway, the knockout of Tcf7l2, which encodes the Tcf4 transcription factor, is lethal due to the lack of intestinal mucosa crypts [12]. The proliferation of ISCs is dependent on the Wnt signaling pathway. The proliferating crypt can be destroyed by deletion of cellular Myc (c‐Myc) which is a Wnt target gene [29]. However, excessive activation of the Wnt signaling pathway leads to enhanced proliferation in the ISC compartment, which ultimately causes intestinal adenomas. Thus, the Wnt signal is critical to the maintenance of ISCs, and its activity is strictly controlled by multiple mechanisms (Figure 2).

#### BMP signaling pathway regulates ISC differentiation

The BMP signaling pathway increases its activity with its ligand expression in intravillous and pericryptal mesenchyme [30]. Bone morphogenetic protein receptor II (BMPRII) combines with BMP ligands and activates BMPRI (Figure 2). The phosphorylated receptor‐regulated Smads subsequently binds to Smad4, is translocated into the nucleus, and modulates target gene transcription [31]. The submucosal region generates antagonists of the BMP signaling pathway, such as Noggin and Chordin, which belong to the transforming growth factor *β* (TGF*β*) superfamily [32].

For ISCs, self‐renewal, proliferation, and replication are suppressed by the BMP signaling pathway, thus preventing the increase of their numbers and fission of crypts and promoting cell differentiation (Figure 2). Correspondingly, in mice with a suppressed BMP signaling pathway, a large number of abnormal crypts appear in their small intestine, indicating that the BMP signaling pathway is capable of preventing intestinal hyperplasia. In Noggin transgenic mice, crypts of mutants with ISCs and proliferating cells were detected in the villus of the intestinal epithelium, which may cause gastrointestinal cancers [30]. Mutated Bmpr1a and Smad4 are present in juvenile polyposis syndrome, a high‐risk adenocarcinoma syndrome [33]. In the case of conditionally knocking out Bmpr1a in mice, the ISC compartments are expanded, ultimately leading to intestinal adenomas. Furthermore, the gene of phosphate and tension homology deleted on chromosome 10 has been reported to mediate the convergence of the BMP and Wnt through the phosphatidylinositol 3‐kinase [34]. Moreover, the BMP signaling pathway has been confirmed to suppress Wnt to control ISC self‐renewal (Figure 2) [35].

Thus, tight control of BMP activation is essential, with low BMP signaling in crypts and higher BMP signaling toward the more differentiated cells in villus [36]. BMP signaling prevents epithelial dedifferentiation, and pathway attenuation through stromal Gremlin1 upregulation was required for adaptive reprogramming in intestinal regeneration [37]. Specific to differentiated cell types, BMP signaling in the intestinal epithelium drives a critical feedback loop to restrain IL‐13‐driven tuft cell hyperplasia [38]. BMP7‐ALK3 also promotes the translocation of mucosal langerhans cell precursors to the epithelium. Langerhans cells are originated from predendritic cells and monocytes [39]. Thus, control of BMP signal raises the possibility of therapeutic pathways in IBD.

#### Notch signaling pathway maintains ISCs and balances secretory and absorptive progenitors

The Notch signaling pathway is critical in regulating the regeneration of intestinal mucosa by participating in the maintenance of IECs and their antibacterial activity [40]. The Notch signal consists of receptors, ligands, and DNA binding proteins (Figure 2). In mammals, Notch receptors (Notch1–4) are distributed widely in lymphocytes, endothelial cells, and IECs with Notch 1 mainly located in the intestine. Membrane‐bound ligands (Jagged1,3 and Delta‐like1,3,4) interact with receptors and tumor necrosis factor alpha (TNF‐*α*)‐converting enzymes in adjacent cells to release the intracellular domain of Notch via *γ*‐secretase‐mediated proteolysis (Figure 2). Soluble Notch intracellular domain moves to the nucleus and regulates gene expression by combining with the transcription factor recombination signal binding protein (RBP) [41].

Notch activation promotes the proliferation of ISCs and determines the differentiation fate of ISCs (Figure 2) [42]. Physiologically, ISCs maintain a steady state via their proliferation and apoptosis. In the case of epithelial damage, substantially more stem cells proliferate for repair and reconstruction compared with a healthy state. Thus, activation of the Notch signaling pathway is required to help repair mucosal damage to the colon. Recently, reduced number of columnar cells and increased death of apoptotic cells that appeared at the bottom of the intestinal epithelium were observed in mice treated with inhibitors of Notch signaling. After 20 weeks, ulcerative colitis (UC) developed [43] and the loss of colonic cortex, inflammatory cell infiltration, and little regeneration of epithelial cells were also observed [40]. In other studies, the Notch signaling pathway promoted the proliferation of intestinal progenitor cells and regulated the immune function of ISCs by increasing the secretion of PLA2G2A in PCs, which ultimately relieves UC [40, 44].

After proliferation, stem cells are transformed into different types of cells via the regulation of the differentiation system. The Notch signaling pathway plays a role in the anaphase of mitosis to influence intestinal progenitor cells. Activation of the Notch signaling pathway promotes differentiation of the absorptive lineage and inhibits the generation of the secretory lineage specification [45]. Deletion of the target gene, RBP‐J [46], and simultaneous repression of Dll1 [47] lead to reduced Lgr5^+^ stem cells and increased GCs. This regulation of ISC development is achieved by expressing Notch target genes, such as hairy and enhancer of split 1 (Hes1) in IECs [48]. Hes1 represses mouse atonal homolog 1 (Math1), which is a central transcription factor for differentiation to secretory lineages [49]. Moreover, knocking out Math1 and Hes1, respectively, has the opposite effect [44]. Hes1 deletion increases secretory cells and decreases absorptive cells in the intestine (Figure 2) [50]. In conclusion, the Notch signaling pathway cooperates with the Wnt signaling pathway to maintain the stemness of ISCs and promote their differentiation into absorptive lineages.

## ISCs AND THEIR RELATIONSHIP WITH INTESTINAL MICROBIOTA: A FEASIBLE PATHWAY TO ALLEVIATE INTESTINAL DISEASES

IBD, including Crohn's disease and UC, is a key factor in the destruction of gastrointestinal homeostasis. To recover from IBD, maintaining intestinal barrier integrity is certainly a feasible strategy [51]. In contrast to other stem cells, ISCs are located at the base of intestinal crypts and coexist with gut bacteria and the mucosal immune system [52]. These three jointly play key roles in epithelial regeneration, pathogen resistance, and inflammatory disease relief (Table 1) [53].

**Table 1:** Effects of specific intestinal microbiota on ISC function

	Effect on ISCs	Animal model or organoid	Reference
*Pseudomonas entomophila*	Interfere with epithelial renewal by microbial virulence and intestinal pathology	Drosophila	[54]
*Pseudomonas aeruginosa*	Induce apoptosis in epithelial cells, following ISC overproliferation	Fly	[55]
Acinetobacter	Provide advantage to the intestinal epithelial, and maintain crypt homeostasis through the expression of particular microbe‐associated molecular patterns or the production of specific metabolites	Human	[9]
*Erwinia carotovora carotovora*‐15	Strongly stimulate ISC division, promoting a rapid turnover of the gut epithelium and a high oral dose induces genes associated with cell growth, wound repair, and the stress response	Drosophila	[56]
	Induce a nonlethal infection	Mice	[57]
*Serratia marcescens*	Induce local intestinal immunity and phagocytosis	Fly	[58]
*Clostridium difficile*	Damage intestinal tissue and block disease recovery through TcdB toxin	HEK293 STF cells, C57BL/6J mice	[59]
*Lactobacilli reuteri*	Stimulate growth and recovery of the gut and intestinal organoids after DSS treatment or TNF‐*α* damage	Organiod, C57BL/6J mice	[60]
	Metabolize raffinose to fructose, which reduces ISC turnover and fuels ISC proliferation	Mice	[61]
*Lactobacilli reuteri* D8	Activate phosphorylation of STAT‐3 by facilitating IL‐22 and further accelerate ISC regeneration, stimulate crypt proliferation, and promote intestinal epithelial recovery	Organiod, C57BL/6J mice	[62]

Abbreviations: DSS, dextran sulfate sodium; HEK293, human embryonic kidney 293; IL‐22, interleukin 22; ISC, intestinal stem cell; STF, Super TOPFlash; TcdB, clostridium difficile toxin B; TNF‐*α*, tumor necrosis factor alpha.

### ISC differentiation affects gut microbes

The proliferation and differentiation of ISCs are jointly regulated by Notch, Wnt, BMP, and other signaling pathways. Notch signal mainly controls the differentiation direction of ISCs. Activated Notch signal results in decreased secretory cells, including PCs, GCs, and EECs, while increased absorptive cell differentiation [45]. Absorptive cells can secrete immunoglobulin A (IgA). Secretory IgA (SIgA) can recognize bacteria and mediate antigen cross‐linking, which is involved in avoiding opportunistic pathogens to enter and disseminate in the systemic compartment; this primary function of SIgA is referred to as immune exclusion. In mice gut, specific IgA antibodies intervention also effectively protected mice against pathogen infections, including *Salmonella typhimurium* [63], *Vibrio cholerae* [64], *Shigella flexneri* [65], and *Helicobacter pylori* [66]. In addition, antigen–SIgA complexes will be recognized by microfold (M) cells. The processed complexes are more readily excreted from the intestinal lumen or presented to dendritic cells to induce an immune response against bacteria [67].

Wnt proteins secreted by PCs can maintain ISC dedifferentiation. PCs are the only differentiated cell type in crypts, which protect stem and proliferative cells through the secretion of α/β defensins, antimicrobial peptides, and so forth [68, 69]. In addition, Wnt signaling can also be stimulated by the binding of Rspo to Lgr‐family members. The Rspo3–Lgr5 axis induces secretory cell differentiation and secretes antibacterial proteins, such as intelectin‐1. Intelectin‐1 binds to and agglutinates *Helicobacter pylori*, impairing its motility [70]. Besides Notch and Wnt, BMP signaling can promote cell differentiation by inhibiting Wnt signaling. TGF*β*/BMP immune signaling affects the abundance and function of *Caenorhabditis elegans* gut commensals. Disruption of TGF*β*/BMP turned a normally beneficial Enterobacter commensal into a pathogen [71]. The intestine harbors multiple microbial communities that closely interact with ISCs. Intestinal microbiota plays an important role in sustaining the physiological state by direct bactericidal effects, competing with pathogens and probiotic action [72]. However, their dysbiosis may result in intestinal imbalance for the host and lead to IBS [73].

### The presence of intestinal microbes and the ISC renewal rate

**Figure 3:**
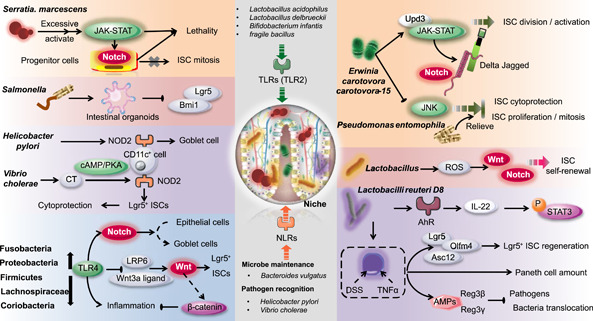
ISCs and their relationship with the intestinal microbiota: a feasible pathway to alleviate intestinal diseases. The intestine harbors multiple microbial communities that coexist and closely interact with ISCs. Microbial communities and ISCs jointly play key roles in modulating intestinal disorders. The intestinal microbiota can affect the mucosa immune system and regulate ISCs. PRRs, especially TLRs and NLRs, regulated by the intestinal microbiota are capable of regulating the ISC niche downstream. Microbial communities involved in this pathway include but are not limited to Fusobacteria, Proteobacteria, Firmicutes, Lachnospiraceae, Coriobacteria, *Helicobacter pylori*, and *Vibrio cholerae*. Bacteria, such as *Serratia marcescens*, *Erwinia carotovora carotovora*‐15, and *Pseudomonas entomophila*, participate in intestinal homeostasis maintaince, injury repair and ISC status via these pathways. AhR, aryl hydrocarbon receptor; Bmi1, B cell‐specific Moloney murine leukemia virus integration site 1; cAMP, cyclic adenosine monophosphate; DSS, dextran sulfate sodium; IL‐22, interleukin 22; ISC, intestinal stem cell; Lgr5, leucine‐rich repeat‐containing G protein‐coupled receptor 5; LRP6, lipoprotein receptor‐related protein 6; NLRs, NOD‐like receptors; NOD2, nucleotide‐binding oligomerization domain 2; PKA, protein kinase A; PRR, pattern recognition receptor; Reg3, antimicrobial‐regenerating islet‐derived 3 lectins; ROS, reactive oxygen species; TLRs, Toll‐like receptors; TNF‐*α*, tumor necrosis factor alpha; Upd, uniparental disomy.

Germ‐free (GF) animals (including sterile Drosophila, zebrafish, mice, and pigs) and intestinal organoids are ideal models to demonstrate the role of indigenous microbes on intestinal and ISC homeostasis. In addition, porcine intestinal organoids have been cultured and are used widely in agricultural, veterinary, and biomedical research [74]. The proliferation of IECs in GF animals is related closely to the intestinal villi. In addition, in the colon of GF animals, a reduced proliferation rate and fewer cells are identified in the crypt compared with the normal animals [75, 76]. Other changes in GF animals, such as reduced activities of the villous capillary network and digestive enzymes, decreasing Peyer's patches and impaired peristaltic activity, may also be observed [77]. However, after colonizing bacteria in GF animals, these changes are reversed to normal levels. Colonization in the gut of GF animals by *Bacteroides thetaiotaomicron* alters the expression of genes associated with intestinal barrier functions, metabolism, angiogenesis, and the nervous system [78]. Intestinal microbes can also activate the immune system in the host. An experiment conducted by Mazmanian et al. [79] showed that the proportion of CD4^+^ T cells was reduced in GF mice. Colonization of GF mice by *Bacteroides fragilis* increased the production of polysaccharide A, which benefited the expansion of T cell populations. In addition, peptidoglycan produced by Gram‐negative bacteria is essential for the genesis of isolated lymphoid follicles [80]. The presence of gut microbes also affects ISCs and the mucosal immune system, which can cause intestinal disorders [81]. Hyperproliferative ISCs and abnormal intestinal morphology appeared in Drosophila with symbiotic bacteria and vanished under GF conditions [56, 82]. However, for zebrafish, the presence or absence of gut microbes caused similar gut defects in the absence of phosphoinositide‐3‐kinase class 3 [83]. Intestinal microbiota can regulate the rate of epithelial cell renewal of the host. Probiotics can help restore imbalance in an injured gut and promote its recovery [84, 85]. Unlike commensal flora, pathogens exert a dose‐dependent effect or epithelial renewal, which is correlated with a certain microbial density [86]. A high lethal dose of pathogenic bacteria infection inhibits epithelial cell renewal [56]. Avirulent or attenuated *Pseudomonas entomophila* affected epithelial renewal by the extent of microbial virulence and intestinal pathology (Figure 3) [54]. After *Pseudomonas entomophila* and *Pseudomonas aeruginosa* infection, apoptosis was induced in epithelial cells, following ISC overproliferation [55].

Early life is an important stage of intestinal development. Expression of erythroid differentiation regulator‐1 (Erdr1) can only be achieved in the presence of the microbiota in early life. Early‐life bacteria govern Erdr1‐mediated Lrg5^+^ ISCs enhancement, epithelial proliferation, and regeneration in response to mucosal damage by inducing Wnt signaling [87]. A group of bacteria that prefer to colonize the crypt was also identified. This flora, such as Acinetobacter, provides optimal signaling to ISCs, which suggests the benefit of these microbiotas on intestinal physiology [9]. Also, via the Janus kinase‐signal transducer and activator of transcription (JAK‐STAT) signaling pathway, microbes are efficient in reducing the epithelial cell turnover, as well as activating the expression of uniparental disomy [56]. This process provides an advantage to the intestinal epithelial and maintains crypt homeostasis.

### Effects of specific intestinal microbiota on ISC function

Regulation of ISCs by intestinal microbes is species specific. Infection of intestinal organoids by Salmonella is accompanied by markedly decreased expression of Lgr5 and Bmi1, which are identified markers of ISCs [88]. *Erwinia carotovora carotovora*‐15 can induce a nonlethal infection in mice [57]. A high oral dose of *E. carotovora carotovora*‐15 induces genes associated with cell growth, wound repair, and stress response (Figure 3) [56]. Genes involved in the host response to Serratia marcescens, which induced general immunity have been identified [58]. In addition, *Clostridium difficile* damages intestinal tissue and blocks disease recovery through a toxin called TcdB [59]. The mechanisms by which pathogens damage ISCs can help find ways to prevent damage or develop new treatments.

Commensal Lactobacilli activated ISCs under a physiological status partially attributed to the production of reactive oxygen species [89], which stimulated ISC self‐renewal [90] and affected the Wnt and Notch signaling pathways (Figure 3) [91]. Many redox sensors are involved in this process [90]. By establishing a coculture system for small intestinal lamina propria lymphocytes and organoids [92], *Lactobacilli reuteri* D8 was confirmed to stimulate the growth and recovery of the gut and intestinal organoids after dextran sulfate sodium treatment or TNF‐*α* damage. This protective effect of *L. reuteri* D8 is consistent with increased regeneration of Lgr5^+^ positive ISCs (higher mRNA expression of ISC markers: Lgr5, Olfm4, and Ascl2) and the number of PCs. Mechanistically, *L. reuteri* D8 enhances the secretion of interleukin 22 (IL‐22), which activates the aryl hydrocarbon receptor (AhR). The activated phosphorylation of STAT‐3 mediated by facilitated IL‐22 could then further accelerate ISC regeneration, stimulate crypt proliferation, and promote intestinal epithelial recovery. Expressions of Reg3*β* and Reg3*γ*, which are antimicrobial peptides, are also increased in this altered microenvironment, which could restrict pathogens and reduce bacterial translocation (Figure 3) [60]. *L. reuteri* can also maintain Lgr5^+^ cell number and stimulate epithelial proliferation by promoting Rspo and thus activate Wnt/*β*‐catenin [62]. Moreover, *L. reuteri* can metabolize raffinose to fructose. Increased fructose constitutes a feedforward metabolic loop, which reduces ISC turnover and fuels ISC proliferation [61]. Commensal microbiota, especially Lactobacillus, are effective at maintaining epithelial homeostasis and repairing intestinal injury. Microbial therapy is a promising pathway for alleviating gut disorders.

## INTESTINAL MICROBIOTA REGULATES ISCS BIOLOGY VIA PRRs

Within the intestine, neutrophils and macrophages recognize and respond to abnormal changes in microbiota via pattern recognition receptors (PRRs) [9]. PRRs, particularly toll‐like receptors (TLRs), and nucleotide‐binding oligomerization domain (NOD)‐like receptors (NLRs) [4] are not limited to sensing pathogenic invasions or epithelial injury. They are also capable of regulating ISCs.

### Microbiota regulation of ISCs through TLRs

TLRs are capable of regulating crosstalk between the host and gut microbes through identified microorganism‐associated molecular patterns of symbiotic bacteria [93, 94]. Status and adapter proteins of TLRs affect microbial composition [4]. Furthermore, an interaction among gut microbiota, TLR signaling, and ISCs may exist via the Wnt and Notch pathways [95].

*Lactobacillus acidophilus* NCFM, *Lactobacillus delbrueckii* subsp. *delbrueckii* TUA4408L, *Bifidobacterium infantis* 35624, and polysaccharide A of the *fragile bacillus* can facilitate TLR2 [4, 96]. Among them, *Lactobacillus rhamnosus* GG acts as a “time release capsule” for lipoteichoic acid. Lipoteichoic acid serves as an agonist of TLR2, binding to TLR2, and inducing the migration of mesenchymal stem cells. This process primes the ISC niche to protect epithelial ISCs [97]. In addition, overexpressed TLR4 and microbiota alterations, including their translocation and enhanced abundance of mucosal‐associated bacteria, coexist in the gut microenvironment. Associated bacteria consist of reduced Fusobacteria and Proteobacteria and increased Firmicutes, Lachnospiraceae, and Gram‐positive Coriobacteriaceae in the colonic mucosa (Figure 3) [98]. The proliferation of ISCs, particularly Lgr5^+^ positive ISCs, is activated by TLR4 in intestinal crypts. The related Wnt signaling, the stimulated Wnt receptor LRP6, and the Wnt3a ligand are also suppressed or blocked by TLR4 [99]. With the alteration of proliferation and apoptosis rates in the crypt due to TLR signaling, intestinal homeostasis changes and results in gut inflammation [100].

ISCs can also express TLR4, and the Wnt/*β*‐catenin is a negative feedback loop that inhibits inflammatory responses triggered by TLRs [101]. Furthermore, TLRs are in a critical position to regulate Notch signaling, for which suppression facilitates the differentiation from epithelial cells to GCs [102].

### Regulation of intestinal microbiota via NLR activation

NOD‐2 is a vital member of the NLR subfamily, which is important for recognizing bacteria and sustaining the intestinal microenvironment [103]. NOD2 is involved in pathogen recognition. Changes in composition and translocation of microbiota also modulate the NOD2 signal [103]. The infection of *Helicobacter pylori*, the maintenance of the *Bacteroides vulgatus* number, and GC function rely on NOD2 signaling [104]. Moreover, NOD2 in CD11c^+^ cells provides access to the adjuvanticity of cholera toxin produced by *V. cholerae* to recognize symbiotic microbes, for which activation is also facilitated through the cyclic adenosine monophosphate/protein kinase A system (cAMP/PKA) (Figure 3) [105].

With stimulation, NOD2 triggers the survival of ISCs (Lgr5^+^ positive ISCs), which results in a strong cytoprotective effect against cell death induced by oxidative stress. Thus, intestinal epithelial recovery depends on NOD2 and can be triggered in the presence of microbial‐derived molecules [9, 53].

## EFFECT OF DIVERSE BACTERIA‐RELATED POSTBIOTICS ON REGULATION OF ISCs

The delicate and complex crosstalk between the host intestine and microbiota is essential for the proliferation and differentiation of ISC, which governs the regulation of epithelial regeneration. Microbial components or metabolites, which we call collectively bacterial‐related products, always serve as significant mediators in this interaction. Bacterial‐related products are small, diffusible factors, such as tryptophan metabolites, short‐chain fatty acids (SCFAs), endotoxins, and peptidoglycans, which can engage host cells and regulate intestinal function [106]. Specific molecules affect important aspects of ISC homeostasis, which may be a potential target to regulate normal physiological functions and diseases of the host, such as IBD, obesity, asthma, and heart disease [107].

### Microbial metabolites of tryptophan maintain ISC homeostasis through AhR

**Figure 4:**
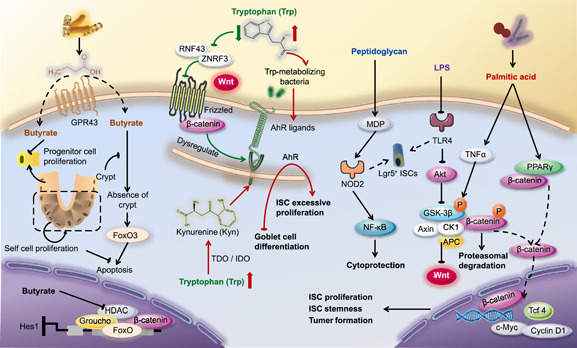
Microbial metabolites regulate physiological functions and intestinal disease by shaping ISCs. Trp maintains ISC homeostasis through AhR. No matter whether Trp metabolites are generated by Trp‐metabolizing bacteria, or Kyn regulated by IDO from Trp, both regulate AhR by serving as AhR ligands and AhR receptor agonists, respectively. SCFAs are identified as ISC effectors. Butyrate, as an inhibitor of HDAC or a ligand for GPCRs, suppress ISC proliferation at physiologic concentrations. However, crypt structure and colonocytes can protect ISCs and progenitor cells to alleviate this effect. Endotoxin is present in the cell wall of Gram‐negative bacteria, which consists of LPS and peptidoglycan. LPS, acting on TLR4, can reduce the proliferation of Lgr5^+^ ISCs or organoids by regulating phosphorylation of Akt, Apc complex, and the Wnt signaling pathway. MDP, enhanced by peptidoglycan, induces proliferation of Lgr5^+^ ISCs and exerts cytoprotection via NOD‐2 and the followed NF‐κB. Bacterial anabolic products, including palmitic acid, activate Wnt signals by both PPAR*δ* and TNF‐*α*. TNF‐*α* promotes GSK‐3*β* phosphorylation and inactivates the Apc complex, which is a negative regulator of Wnt signals. AhR, aryl hydrocarbon receptor; Akt, protein kinase B; Apc, adenomatosis polyposis coli; CK1, Casein kinase 1; c‐Myc, cellular Myc; GPR43, G protein‐coupled receptor 43; GSK‐3*β*, glycogen synthase kinase‐3 beta; HDAC, histone deacetylase; Hes1, hairy and enhancer of split 1; IDO, indoleamine 2,3‐dioxygenase; ISC, intestinal stem cell; Kyn, kynurenic acid; LPS, lipopolysaccharide; MDP, muramyl dipeptide; NF‐κB, nuclear factor kappa B; NOD, nucleotide‐binding oligomerization domain; PPAR*δ*, peroxisome proliferator‐activated receptor δ; RNF43, Ring Finger Protein 43; SCFAs, short‐chain fatty acids; Tcf4, T‐cell factor 4; TDO, tryptophan‐2,3‐dioxygenase; TLR4, Toll‐like receptor 4; TNF‐*α*, tumor necrosis factor alpha; Trp, tryptophan; ZNRF3, Zinc And Ring Finger 3.

AhR was originally classified as an environmental sensor but now is described as the “gatekeeper” in IECs [108, 109]. Nutrition and gut microbiota are interlinked. High dietary tryptophan acts as a stimulator that promotes the growth of tryptophan‐metabolizing bacteria and the generation of AhR ligands, which drive beneficial functions, such as immune homeostasis, intestinal barrier functions, and ISC function (Figure 4).

A vital role of AhR activation is to control the excessive proliferation of ISCs. A dysregulated AhR pathway consistently causes aberrant ISC proliferation in elderly mice. Mechanistically, AhR dysregulation in the gut interferes with the regulation of Wnt‐*β*‐Catenin signaling. This signaling pathway fulfills a critical role in the renewal, differentiation, and maintenance of ISCs and is tightly modulated by Ring Finger Protein 43 (RNF43) and Zinc And Ring Finger 3 (ZNRF3) [109, 110].

As a consequence of dysregulation, AhR‐deficient epithelium leads to enhanced inflammation‐induced tumorigenesis and compromised ability of ISC to regenerate and differentiate. With no capacity to cope with epithelial damage, inflammatory processes proceed and colorectal cancer forms. Fortunately, after supplementing with microbial‐generated AhR ligands, regulation of Wnt‐*β*‐Catenin signaling can be restored and progression of tumorigenesis can be paused [109].

In the gut, the three major tryptophan (Trp) metabolism pathways generate serotonin, kynurenine, and indole derivatives and are carried out under the direct or indirect control of the microbiota [111]. Gut microbiota expressing tryptophanase, such as *Escherichia coli*, *Symbiobacterium thermophilum*, *Peptostreptococcus russellii* [112], *Vibrio cholerae*, *Chromobacterium violaceum*, *Lactobacillus* spp. [113, 114], *Acinetobacter oleivorans*, *Serratia marcescens* [115], and *Pseudomonas chlororaphis* metabolize Trp into indoles. Indoles and indole derivatives, such as indole‐3‐aldehyde, indole‐3‐acid‐acetic, indole‐3‐propionic acid, indole‐3‐acetaldehyde, and indoleacrylic acid are important signaling molecules [116]. These molecules activate AhR and mediate the regulation of AhR on ISCs. In addition, when induced by proinflammatory cytokines, the kynurenine pathway primarily mediates tryptophan metabolism. This process is regulated by indoleamine 2,3‐dioxygenase (IDO) in the mammalian gut to generate kynurenine, which acts as a ligand and a receptor agonist for AhR [4, 115]. With the participation of kynurenine and IDO‐1, activation of AhR moderately induced GC differentiation. Moreover, kynurenine was also confirmed to promote GC differentiation under regulatory signals from Hes1, Hath1, Wnt, Notch, and AhR [117].

### Effects of SCFAs on ISC homeostasis

The relationship between bacterial metabolites and intestinal functions is well established. For example, SCFAs are important energy sources and signaling molecules for the epithelium [118]. Researchers have identified several SCFA receptors [119], and their potential regulatory pathways are involved in nutrient metabolism and ISC proliferation and differentiation [120]. High expression of G protein‐coupled receptor 43 (GPR43) in neutrophils and eosinophils recognizes SCFAs and establishes a connection between SCFAs and the immune system [121]. Butyrate is an inhibitor of histone deacetylase or a ligand for GPCRs and can affect the intestinal barrier and modulate stem cell activity by inhibiting the proliferation of intestinal progenitor cells under physiologic concentrations (Figure 4) [122, 123].

ISCs can produce butyrate when butyrate generated by beneficial microbes interferes [8, 124]. As the intestine attempts to repair damage or injury, the proliferation of ISCs is suppressed due to butyrate, thereby leading to delayed recovery of intestinal tissue [8]. A recent study further supports the notion that crypt structure contributes to the regulation of butyrate and ISCs [8]. Differentiated colonocytes produce butyrate, which may lead to crypt‐loss, suppressed proliferation, and delayed wound repair, possibly to prevent butyrate from touching proliferating ISCs in crypts. Inhibition of butyrate depends on forkhead box O3 (Foxo3). The crypt structure of mammals protects ISC proliferation because colonocytes serve as a metabolic barrier, which can consume butyrate [8].

### Relationship between bacterial cell wall components and ISCs

Peptidoglycans are a major component of bacterial cell walls. Serving as an immune enhancer, peptidoglycans may be used to enhance disease resistance. Natural extracts of peptidoglycans are limited in clinical applications. The active peptidoglycan, muramyl dipeptide (MDP), has strong immunological potential. MDP induces the proliferation of Lgr5^+^ ISCs and prevents apoptosis under oxidative stress. Due to the activation of NOD2, this protection provided by MDP only occurs in the case of injury (Figure 4) [53]. The cytoplasmic level of NOD2 in Lgr5^+^ ISCs is substantially higher than that in PCs. NOD2 can perform cytoprotection following activation by MDP without regulation of PCs [53].

Endotoxin is present in the cell wall of Gram‐negative bacteria, and its main component is lipopolysaccharide (LPS). LPS is a modulator of TLR4 and can reduce the proliferation of Lgr5^+^ ISCs or organoids in mice (Figure 4) [125]. In TLR4 knockout mice, LPS does not show an inhibitory effect on intestinal cells. However, this phenomenon can be reversed by the activation of β‐catenin. Activated TLR4 reduces phosphorylation of protein kinase B (Akt) in epithelial cells of neonatal mice. Akt is the negative regulator of GSK‐3β phosphorylation. As a result, the intact Apc complex leads to the degradation of β‐catenin and a reduction of cell proliferation [126]. However, overexpression of TLR4 activates Wnt/β‐catenin signals, which leads to colonic epithelial cell proliferation, deeper crypts, and progressive development of tumors [127].

ISCs can express PRRs, such as TLR4 and NOD2, and can be activated by ligands with opposite effects. LPS activates TLR4 and induces ISC death. However, MDP protects ISCs via NOD2. Both LPS and MDP exist in the intestine and block each other's effects in a healthy body.

### The relationship between ISC and saturated fatty acids

Palmitic acid is a type of saturated fatty acid distributed in plants and animals. Studies proved that palmitic acid is synthesized by bacteria during fat production. Palmitic acid accompanied with the peroxisome proliferator‐activated receptor (PPAR)δ agonist can improve the number of ISCs and TA cells and can increase the efficiency of their regenerating organoids. Knocking out intestinal PPAR*δ* has no effect on ISCs and TA cells compared with the control but the function of palmitic acid and the PPAR*δ* agonist is inhibited. The PPAR*δ* agonist can move β‐catenin of ISCs and TA cells into the nucleus and activate the expression of its target genes, resulting in cell proliferation and potentially tumors [53]. Palmitic acid can increase the concentration of colonic TNF‐α. TNF‐α can induce GSK‐3β phosphorylation and reduce the decomposing effect of the Apc destruction complex on phosphorylation and degradation of β‐catenin. This mechanism is completed with more translocation into the nucleus and then promotes the expression of c‐Myc and cyclin D1, which are Wnt target genes [128], leading to enhanced proliferation of colonic stem cells (Figure 4). Palmitic acid, a major component of high‐fat diet, increases the number of ISCs and progenitors characterized by stemness or tumor formation. Correspondingly, cells that can be transformed to initiate tumors are also increased in quantity [129].

## CONCLUSIONS

Intestinal homeostasis depends on the dynamic balance between ISC self‐renewal and differentiation. Factors including the ISC niche, intestinal microbiota, metabolites, and endocrine and immune systems coregulate the physiological activity of ISCs. In this review, we focus on targeting the ISC niche for disease therapy and our current understanding of the role, regulation, and intervention of microbiota in this process. Although knowledge of intestinal epithelial mechanisms has made rapid progress in the past few years, characteristics of the tissue are still confusing. How do ISCs in the niche protect the host against intestinal diseases? How do microbes colonize in the gut affect cell differentiation, proliferation, and ISC fate in the intestine? How do endogenous and exogenous factors interact with ISCs and microbes to jointly affect intestinal homeostasis? These and other questions need to be answered. Thus, this review witnesses major advances in ISC niche research, and the complex crosstalk between microbiota and ISCs, which has the potential to be the therapeutic target intimately involved in intestinal diseases. Considering that the detailed interaction of intestinal microbiota and ISCs remains puzzling, we hope the content in this review can fill the gap and provide a useful and topical contribution to the ISCs field. Furthermore, these major advances collectively drive our clinical breakthroughs in regenerative medicine and cancer therapy through microbial transplantation.

## AUTHOR CONTRIBUTIONS

The review was mainly conceived and designed by Xi Ma. Literature was collected by Ning Ma. The manuscript was mainly written by Ning Ma and edited by Xiyue Chen, Lee J. Johnston, and Xi Ma. Xi Ma resourced the project. All authors contributed to, read, and approved the final manuscript.

## CONFLICT OF INTEREST

The authors declare no conflict of interest.

## Data Availability

No new data and scripts were used in this paper. Supplementary materials (figures, tables, scripts, graphical abstract, slides, videos, Chinese translated version, and update materials) may be found in the online DOI or iMeta Science http://www.imeta.science/.
